# Brain fog and non-coeliac gluten sensitivity: Proof of concept brain MRI pilot study

**DOI:** 10.1371/journal.pone.0238283

**Published:** 2020-08-28

**Authors:** Iain D. Croall, Nigel Hoggard, Imran Aziz, Marios Hadjivassiliou, David S. Sanders

**Affiliations:** 1 Department of Infection, Immunity & Cardiovascular Disease, University of Sheffield/INSIGENO, Sheffield, United Kingdom; 2 Academic Unit of Gastroenterology, Royal Hallamshire Hospital, Sheffield Teaching Hospital NHS Foundation Trust, Sheffield, United Kingdom; 3 Academic Departments of Neurosciences and Neuroradiology, Sheffield Teaching Hospitals NHS Trust, Sheffield, United Kingdom; Nathan S Kline Institute, UNITED STATES

## Abstract

**Aims:**

Non-Coeliac Gluten Sensitivity (NCGS) is poorly understood, particularly in terms of its neurological outcomes. We initially conducted a prospective postal survey to investigate its neurological presentation and symptom course. Results from this then motivated a follow-up pilot study utilising brain MRI to characterise potential diagnostic biomarkers for future research.

**Methods:**

Patients with NCGS were recruited from a specialist centre and completed a prospective postal questionnaire (N = 125). This summarised symptoms experienced, their severity and their course. Onset time was compared by Chi-squared analysis to data from the same centre concerning coeliac disease patients (N = 224). Five respondents on a strict gluten-free diet who self-reported brain fog then attended a pilot study, completing MR brain imaging/questionnaires before/after a gluten challenge. “Baseline” data were assessed for abnormalities, while symptom severity and cerebral blood flow (CBF) were compared before/after challenge.

**Results:**

Survey participants were aged 47 (85% female). Prevalence of neurological symptoms were: headaches (51%), brain fog (48%), balance issues (31%), tingling (19%). Median symptom resolution time was 48 hours, while onset was 90 minutes; onset pattern was not significantly different compared to CD patients (p = 0.322). Extra-intestinal symptoms worsened by 37%(±28) during a typical reaction. Predominantly non-statistical observations from the brain imaging study are discussed.

**Conclusions:**

Neurological symptoms in NCGS are common, and onset time is comparable to that in CD. Brain imaging may be a useful future means of investigating physiological injury and responses to gluten in further study.

## Introduction

Non-coeliac gluten sensitivity (NCGS) describes people who self-report gastrointestinal symptoms after ingesting gluten, but do not have coeliac disease (CD) or wheat allergy [1]. NCGS is thought to affect approximately 10% of the general population [2]. Symptoms resolve on a gluten-free diet (GFD) and are similar to what is seen in CD, largely revolving around gastrointestinal discomforts but also with extra-intestinal involvement. Neurological symptoms such as headache and brain fog have been reported [2, 3].

The physiological basis of NCGS is poorly understood. People who follow a GFD for “lifestyle” reasons [4, 5], and the overlap between NCGS and conditions such as IBS adds heterogeneity into the population [5–7]. However, studies have shown evidence of a physiological reaction in patients [8–10], including raised levels of gluten-specific, gliadin antibodies (AGA) [11]. Notably, a meta-analysis of randomised, placebo controlled trials [12] has shown an overall effect in favour of NCGS subjects experiencing symptoms when trials are restricted to those which follow the “Salerno” expert criteria [13]. As current tools such as AGA tests do not perform well diagnostically [14], the Salerno group have also highlighted the need for studies to identify other biomarkers for the condition.

To date, no study has directly focused on the aforementioned neurological symptoms in “classic” (i.e. gastrointestinal) NCGS. The physiological basis of these symptoms may offer new brain-based biomarkers, which would complement others focused on the gut and immune response [13] for use in future trials. Potential candidates for these may be similar to those already reported for CD which would include pathologies such as white matter lesions [15] or altered cerebellar biochemistry [16], as well as “dynamic” variables such as cerebral blood flow (CBF) [17] which may change by measurable degrees alongside symptom onset following a gluten challenge. Brain imaging has never been conducted on classic NCGS patients, meaning pilot data would be very valuable.

Deeper investigation of other, broader NCGS features would also assist planning of future trials. These include the prevalence of NCGS patients who self-report feeling at least 30% worse after eating gluten, which is a one of the Salerno criteria and may therefore be an important inclusion criteria. Symptom course is also poorly understood, with no available studies reporting symptom onset time in adults within a period of less than 6 hours [3, 18]. These details are important for the planning of experiments which may seek to measure a physiological response following a gluten challenge, as onset times of one hour compared to six hours carry very different implications for the feasibility of study visit plans.

### Aims

We conducted a postal questionnaire of patients who have been diagnosed with NCGS following referral to a specialist CD clinic, characterising overall symptom course, as well as the frequency of neurological symptoms and their severity. This motivated follow-up pilot study on a group of respondents to perform the first brain imaging experiment of NCGS.

## Methods

### Participants

Participants comprised patients who had previously been referred to a specialist gastroenterology celiac clinic in Sheffield (UK) after self-reporting gluten sensitivity. All patients had gluten sensitivity but had completed clinical investigations which ruled out CD (HLA-DQ2 & DQ8 tested, gluten challenge of 10g gluten daily for 6 weeks followed by a gastroscopy, duodenal biopsy and antibody testing for TTG, EMA and IgA level). Patients were initially contacted by letter in February of 2018, inviting them to complete an accompanying survey. A pool of 221 patients were approached, and 125 returned completed questionnaires. Ethics for the questionnaire were approved via the North Sheffield REC.

### Questionnaire

The current analyses focus on a subset of responses given to a larger postal questionnaire, which overall took ten minutes to complete. Regarding the present study, demographic information and answers to a series of bespoke questions were collected. These assessed what symptoms are experienced during a typical gluten reaction; the multiple-choice symptom options were based on those given in the Reactions Experienced After Consuming Gluten (REAC-G) [19] tool, although this was modified to include additional neurological outcomes as available options (brain fog, feeling off-balance & tingling sensations). The REAC-G was also replicated to ask about symptom onset and resolution time (responses to these questions were given as a “free text” entry). Finally, visual analogue scales based on Salerno criteria guidance [13] assessed symptom severity. To consider the known variation in presentation experienced by NCGS patients, this was done twice to separately estimate the magnitude of overall intestinal / extra-intestinal symptoms.

### Overview of available serological information and control reference data

As part of their initial clinical investigations many NCGS patients had also been tested for gliadin antibodies (AGA). The kit used for this differed based on the date of their care, as the local hospital trust switched the ELISA assay to “Phadia 2500” in 2015 [20]. The study team have collected data of AGA incidence in healthy volunteers using the same method for the purposes of kit calibration; at the time of writing this has been completed in 96 people without any known gluten sensitivity. The AGA positivity rate (IgA or IgG) in NCGS survey respondents who were assessed with the current kit was ascertained for comparison to the rate in these healthy volunteers.

### Pilot MRI study

Respondents who had indicated on the survey that they were on a strict gluten-free diet, that brain fog was a symptom they experienced and that their symptoms usually take less than 2 hours to begin following gluten ingestion were invited to the pilot study. Further criteria were then applied when discussing joining; no history of diagnosed neurological or psychiatric conditions (except for depression), no history of drug/alcohol misuse, not currently taking any psychoactive medication, not currently participating in any other research studies and no contraindications to MRI scanning. Twenty-nine potential subjects were approached before 5 were recruited. Ethics for the pilot scanning study was approved by the Yorkshire and the Humber REC.

The overall design of the imaging experiment involved a baseline scan, followed by a gluten challenge, followed by a second brain MRI scan. Immediately before each scan, visual analogue scales measuring anxiety, depression, headache, “clear-headedness” and overall healthstate symptoms were given. The Hospital Anxiety and Depression scale (HADS [21]) was also completed once in the pre-challenge testing session, also before that scan.

### Gluten challenge

Subjects chose their own gluten challenge, which they ate under the supervision of a study team member. All participants chose to eat a single sandwich which was either bought from a store, or made at home using store-bought bread. Though there will be variability within this, it has been previously estimated that two slices of wheat bread contain approximately 3g of gluten [22].

### Imaging and clinical reporting of results

Imaging was performed on a 3T Philips Ingenia scanner. Sequences were chosen that are effective at investigating for specific phenomena which have been previously reported in CD. These included T1-weighted (T1), FLAIR, Magnetic Resonance Spectroscopy (MRS) of the vermis (a grey matter region of the cerebellum) and Arterial Spin Labelling (ASL) scans.

Assessment of FLAIR and MRS scans were intended to replicate types of routine clinical investigation. The FLAIR scans were assessed by a consultant neuroradiologist for white matter lesions of a clinically suspicious level (with respect to the patient age). The N-Acetylaspartate/Creatine (NAA/Cr) ratio was determined from the MRS scan and compared to cut-off points to determine abnormal levels, also replicating local clinical protocols. These cut-offs are locally calibrated with respect to healthy control data previously collected from the same scanner, and are set as 2 standard deviations below that control mean. ASL data were used to calculate grey matter CBF before / after gluten challenge.

Full information about image acquisitions and processing are available in the S2 File.

### Data analysis

All analyses were conducted using SPSS.

#### Postal survey

For the postal survey, variables concerning symptom-type frequency, symptom onset/resolution time and symptom severity were summarised. Symptom onset time was converted to categorical increments which matched those in a previously reported study from the same centre which focused on symptom onset time in a population of biopsy-proven CD patients (N = 224; [23]); the pattern of onset was then compared with this CD data by Chi-squared analysis. AGA positivity in respondents who were tested with the current kit was compared by Chi-squared testing to the rate of positivity in healthy volunteer data. Testing was conducted with the maximum amount of survey data available per-analysis.

#### Imaging study

The imaging study is included and intended as a pilot analysis for “observational” interest in providing the first data of this kind. This data for the most part was not subjected to statistical testing; results from MRI scanning were summarised in terms of evidence of white matter disease and abnormal MRS results according to clinical protocols, while participants who scored above conventional cut-offs to indicate either depression or anxiety on the HADS scale were also identified. In terms of the gluten intervention, percentage change in grey matter CBF between the pre- and post-gluten acquisitions was calculated, and change in symptom scores as measured by visual analogue scales was compared by paired t-test.

## Results

### Postal survey

The mean age of the 125 respondents was 47 years (range: 18–78, SD = 14) with 84.8% being female. Of the respondents, 39 had been tested for AGA using the current assay with 7 being found positive (17.9%). Data collected in 96 healthy volunteers as part of calibration work for the same kit has overall shown 12 to be positive (12.5%). This is not significantly different by X^2^ comparison (*p* = 0.409).

**Fig 1:**
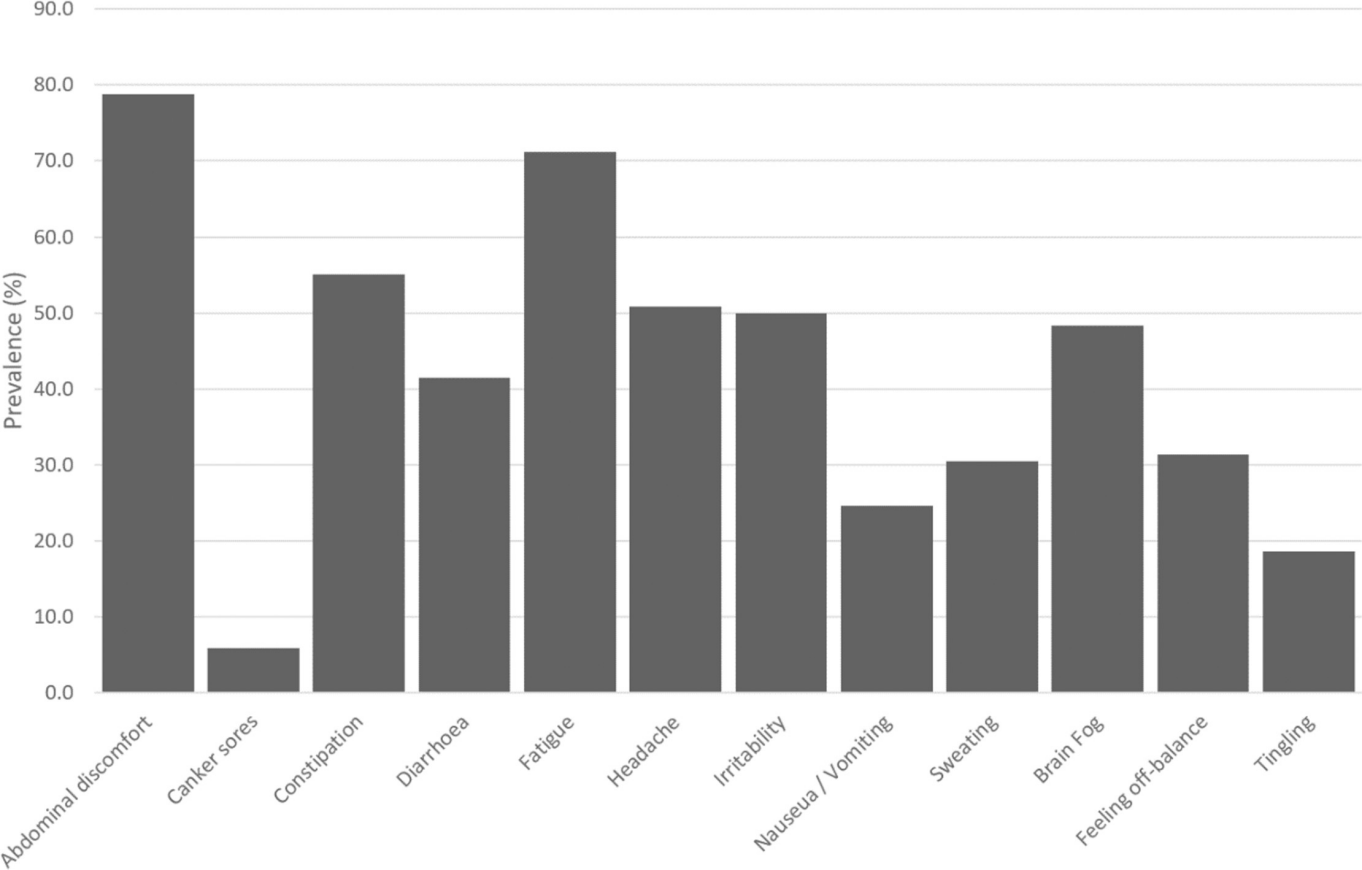
A bar chart showing the percentage prevalence of different symptoms in gluten reactions from the postal survey. “Canker sores” is synonymous with mouth ulcers, i.e. oral aphthae.

The most common intestinal symptom was abdominal discomfort (78.8% of subjects), while the most common extra-intestinal one was fatigue (71.2%). Of note, headache was reported in 50.8% of subjects, brain fog in 48.3%, feeling off-balance in 31.4% and tingling sensations in 18.6%. All responses are shown in Fig 1.

The mean(SD) overall severity of symptoms while suffering a reaction (where 0% represents no discomfort and 100% represents maximum discomfort according to the visual analogue scale responses) was 74.1%(21.9) for intestinal symptoms and 70.3%(27.6) for extra-intestinal symptoms. This represented a mean increase in severity of discomfort (when compared to the gluten-free baseline) of 45.3%(24.4) for intestinal symptoms and 36.5%(27.5) for extra-intestinal symptoms. The fraction of participants who estimated they felt more than 30% worse during a reaction was 75.9% for intestinal symptoms and 61.1% for extra-intestinal symptoms. Overall, the fraction of participants for who reported at least one of these phenotypes to worsen by over 30% was 83.3%.

**Fig 2:**
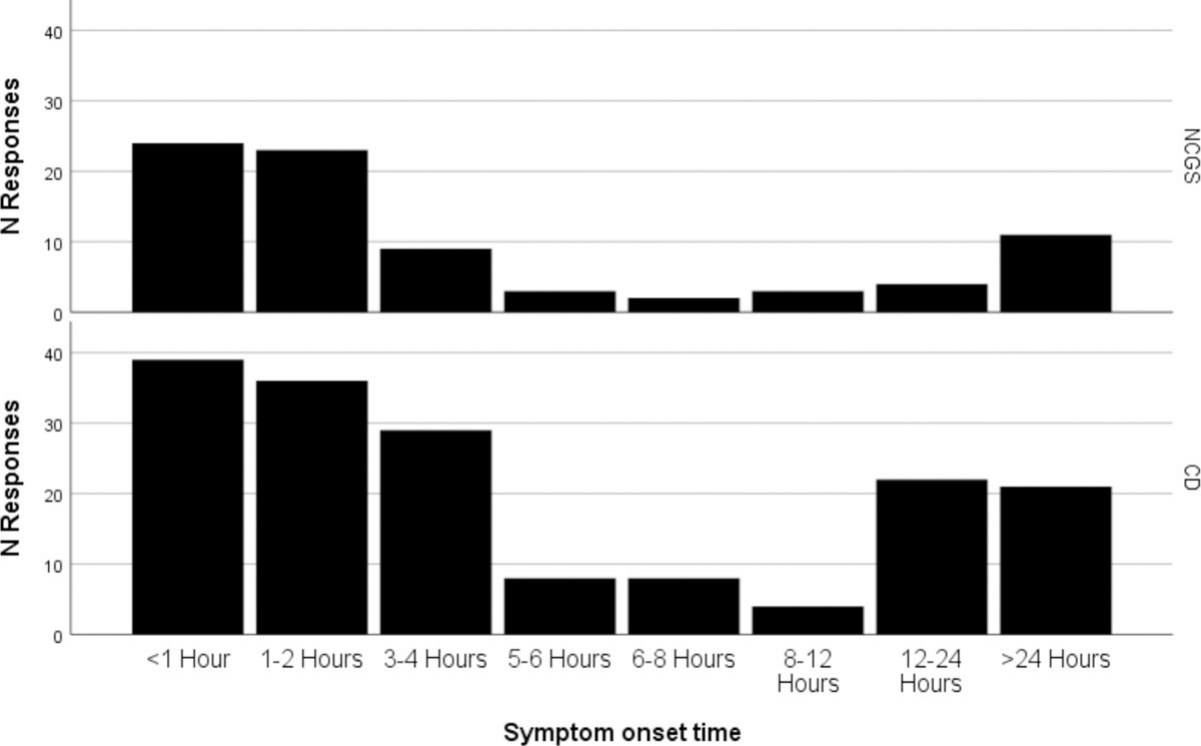
Histogram showing the overall pattern of symptom onset time after eating gluten, compared between the current NCGS postal survey population and previously-collected data from CD patients.

Median symptom onset time was 90 minutes; 44.9% of participants reported that symptoms occurred in an hour or less, while 87.0% reported them to start within 12 hours. Median symptom resolution was 48 hours, with 78.0% of participants reporting that symptoms resolved within a week. Chi-squared analysis comparing symptom onset time to previously collected/reported data from CD patients (N = 167) showed no significant difference (p = 0.322, see Fig 2).

### Pilot imaging study

Demographic information for the five subjects who attended this pilot study are shown in Table 1, alongside “baseline” observations variables with respect to their clinical normality. Reports based on their FLAIR images indicated three had suspicious levels of white matter lesions, while NAA/Cr values of the vermis were found to fall below the cut-off in two of these subjects. Using conventional cut-offs, HADS scores indicated one subject to be “borderline” for both depression and anxiety (i.e. scored between 8–10), and another subject met full criteria for anxiety (i.e. score >10). Each of these subjects had also exhibited either abnormal white matter of NAA/Cr findings.

**Table 1:** Demographic and baseline characteristics of the subjects who attended the imaging pilot study. The NAA/Cr cut-off for abnormal readings in ≤0.95. HADS cut-offs are according to convention where <8 is a “non-case”, 8–10 is a “doubtful case” and >10 is a “case”.

Subject	*Age*	*Sex*	*White Matter Report (bold if abnormal)*	*NAA/Cr result (bold if abnormal)*	*HADS—Depression score (bold if abnormal)*	*HADS—Anxiety score (bold if abnormal)*
*1*	*33*	*F*	***Abnormal***	*0*.*97*	***9***	***9***
*2*	*37*	*F*	*Normal*	*1*.*00*	*3*	*4*
*3*	*68*	*M*	***Abnormal***	***0*.*95***	*2*	*6*
*4*	*32*	*F*	***Abnormal***	***0*.*87***	*4*	***11***
*5*	*33*	*F*	*Normal*	*1*.*05*	*0*	*1*

Table 2 shows results pertaining to the gluten challenge. Symptom scores before / after gluten (where a higher score always indicates a worse outcome) were not significantly different for feelings of anxiety, depression or overall healthstate. These scores also did not show a consistent direction of change. However, headache (before; 7.91±5.73, after; 29.79±17.09, p = 0.027) and brain fog (before; 19.58±30.16, after; 50.83±32.22, p = 0.042) scores significantly and consistently worsened across all subjects. ASL imaging to explore any changes in grey matter CBF due to gluten did not show a consistent pattern of change; grey matter perfusion values for four of the five subjects variably rose or fell by magnitudes of approximately 10% or less. However, of potential note CBF for the remaining subject was recorded as being 68.4% higher after eating gluten.

**Table 2:** Data pertaining to the gluten challenge portion of the pilot analysis. Symptom change scores are percentages derived from visual analogue scales administered before and after the gluten, and a higher value always indicates a worse outcome; headache and brain fog both changed significantly across the group. CBF values are given in units of ml/min/100g.

*Subject*	*Anxiety change*	*Depression change*	*Headache change*	*Brain fog change*	*Overall unwellness change*	*Baseline GM CBF*	*Follow-up GM CBF*	*CBF % change*
1*	*-3*.*1%*	*-32*.*3%*	*+17*.*7%*	*+20*.*8%*	*+17*.*7%*	*34*.*5*	*31*.*4*	*-8*.*9%*
*2*	*-15*.*6%*	*+2*.*1%*	*+1*.*0%*	*+10*.*4%*	*+9*.*4%*	*27*.*8*	*46*.*8*	*+68*.*4%*
*3*	*+4*.*2%*	*+4*.*2%*	*+19*.*8%*	*+26*.*0%*	*+30*.*2%*	*36*.*2*	*36*.*6*	*+1%*
*4*	*-57*.*3%*	*-64*.*6%*	*+34*.*4%*	*+71*.*9%*	*-12*.*5%*	*38*.*5*	*43*.*4*	*+12*.*8%*
*5*	*+2*.*1%*	*+1*.*0%*	*+36*.*5%*	*+27*.*1%*	*+27*.*1%*	*41*.*4*	*36*.*6*	*-11*.*6%*

*All subjects except #1 completed both testing sessions on the same day; subject 1 completed baseline and gluten-challenge sessions 18 days apart.

## Discussion

This study combined a postal survey with a pilot brain imaging analysis to help characterise features of NCGS which may assist in future research trials, particularly those which may wish to focus on neurological outcomes.

There is a need for diagnostic biomarkers of NCGS [13]. There is limited research showing AGA is raised in NCGS patients compared to the general population [11]. In the current study the positivity rate in patients was 17.9%, compared to 12.5% of healthy volunteers, (a non-significant difference). This generally supports previous literature showing that it is not overall a strong measure for diagnosing conventional NCGS [14]. Future research may still benefit from investigation of how gliadin positivity may interact with neurological outcomes specifically, as these antibodies have been indicated as potentially harmful for the brain [24].

The brain has been shown to be affected in a number of gluten-related disorders, from blunt neurological conditions like gluten ataxia [25] to “classical” CD [16, 17, 26, 27]. In the current study neurological symptoms during typical gluten reactions of “classic” NCGS patients were reported at high rates, including headaches and brain fog each in approximately half of the group, as well as feeling off-balance in a third of subjects and sensory symptoms in a fifth. Symptom severity was reported to increase markedly and by similar amounts when comparing intestinal and extra-intestinal outcomes; overall approximately four fifths of patients felt at least 30% worse in one of these subtypes. This indicates they likely meet the Salerno criteria [13] for inclusion in randomised trials.

Median time of symptom onset was reported as being 90 minutes; almost half of participants experienced symptoms within the first hour. Median symptom resolution time was 2 days, with just over three-quarters of patients recovering within a week. The pattern of onset time was not significantly different to that in CD patients from the same centre. NCGS and CD are known to be very similar in terms of their symptomatic presentation, and this draws further attention to the high degree of overlap between the conditions. It also shows that it is feasible for future studies to attempt physiological measurements of response to gluten challenges during the same study visits.

These findings, indicating overall that neurological features in NCGS may be very relevant to the pathophysiology of the condition, motivated a pilot analysis to perform the first ever brain imaging of NCGS patients. This was completed on five participants, and is presented as a pilot study so that these methods may be introduced in this context. The purpose of pilot studies are to “examine the feasibility of an approach that is intended to be used in a larger scale study” [28], rather than being a full and formal analysis. Discussion presented here is given with the purpose of introducing potential future neurological biomarkers for such studies, which may also seek to include control groups or other NCGS patient subgroups, and is not a set of “formal” findings or interpretations.

In replications of routine clinical protocol, suspicious quantities of white matter lesions (N = 3) and low vermis NAA/Cr ratios (N = 2) were reported. These were each investigated as other research has indicated these may hold relevance to other gluten-related disorders. White matter lesions are a non-specific brain pathology known to occur during normal aging [29]. However, they appear with greater size and frequency in a number of conditions such as vascular dementia [30] and multiple sclerosis [31], and have also been reported as an outcome in CD [16] (potentially linking with the increased risk of vascular dementia which CD is known to carry [32]). Similarly, low NAA/Cr is also known to be present in CD [17] and other neurological gluten conditions such as gluten ataxia [33]. These values were found to fall below clinical cut-offs in two of the current subjects and were therefore flagged as potentially abnormal. These cut-offs are the same as used clinically, and are set as being two standard deviations below the mean values of healthy controls, imaged with the same sequence on the same scanner as part of local calibrations. Accompanying these imaging results, the Hospital Anxiety and Depression Scale also determined one subject met full criteria for anxiety, and another to be “borderline” for both anxiety and depression. Depression and anxiety have been shown to be comorbidities of CD [34].

The imaging study also involved an interventional component whereby participants completed other assessments before and after a gluten challenge. Feelings of brain fog and headache each worsened in a consistent pattern and by an overall significant magnitude after eating gluten, although the lack of a placebo-controlled design means this should be interpreted with caution. A repeat MRI scan also measured CBF. The method used to study this, “ASL”, is popular within neuroimaging research [35, 36], but does suffer from relatively high levels of noise [37]. This is important to consider as only very marked differences in CBF should be considered potentially interesting. Four of the five subjects showed neither a “large” or consistent CBF change, however for the final participant CBF increased by 69% after eating gluten. While this may hypothetically represent a dynamic physiological response to the gluten challenge, a number of factors outside of the control of the study design can influence CBF over the course of a day [38].

The study has a number of limitations. Patients who may be sensitive to causative agents other than gluten, such as FODMAPs, were not directly controlled for. However, this possibility will have been some part of the initial clinical investigations for the patients and therefore the likelihood of this should have been reduced by some degree. As already discussed, the imaging study is also significantly limited by its small sample size, and aspects of its interventional component are additionally confounded by the possibility of “nocebo” effects (although this should not influence the CBF readings, as they are physiological measurements). As stated, it is not appropriate to consider this part of the paper a full, formal scientific analysis, and discussion around those findings is only intended to indicate potentially interesting future research avenues.

## Conclusions

A combined postal survey and brain imaging analysis revealed that “typical” NCGS frequently involves neurological symptoms, of non-trivial severity, which may be studied within 2 hours following gluten ingestion. The first neuroimaging data of its kind characterised a number of clinical variables which may be useful variables in future trials studying the pathophysiology of NCGS.

## Supporting information

S1 File (SAV)

S2 File (DOCX)
