# Supplementary material for: Brain fog and non-coeliac gluten sensitivity: Proof of concept brain MRI pilot study
Source: PLoS One. 2020 Aug 28;15(8):e0238283. doi: 10.1371/journal.pone.0238283 (PMC7454984; doi:10.1371/journal.pone.0238283)
Supplement: S2 File — (DOCX) [file pone.0238283.s002.docx]

**Information concerning MRI acquisition parameters and analysis**

Sequences included in the study were:

**T1-Weighted (T1)** - These are standard “structural” MRI scans that are ideal for showing the brains anatomy with good image contrast between grey and white matter tissue. The major acquisition parameters for these are: *3D “MPRAGE”, 1x0.94x0.94mm resolution, TR/TE=8.19/3.79ms*

**FLAIR** - These are similar to T1W scans in that they are also “structural” but the image contrast in them is most effective at highlighting “white matter lesions”, which are a common type of brain pathology reported in conditions such as dementia (and have been previously reported in CD[1]). The major acquisition parameters for these are: *3D, 0.56x0.94x0.94mm, TI/TR/TE=1650/4800/290ms*

**Magnetic Resonance Spectroscopy (MRS)** - A single-voxel MRS scan quantifies the concentration of metabolites in the specific part of the brain the voxel is placed. These metabolites are used clinically in the monitoring of gluten ataxia patients^4^, and have been shown to be affected in CD[2]. The major acquisition parameters for these are: *Single voxel, 1H PRESS sequence, 20x10x20mm resolution, placed in the vermis of the cerebellum*

**Arterial Spin Labelling (ASL)** - ASL scans are “quantitative” scans whose purpose is to calculate cerebral blood flow, which has been shown to be affected in CD[3]. The major acquisition parameters for these are:

- ASL data: *“pcASL” sequence, 2D single-shot “EPI” images, 3x3x8mm resolution, 17 slices, 30 control / label pairs, label distance=96.86mm, label duration=1800ms, post-label delay=1800ms, slice delay=55.88ms, TR/TE=4500/14.38ms*
- M0 data: *2D single-shot “EPI”, 3x3x8mm resolution, TR/TE=10000/120ms*

The first scan involved all of these, while the second scan repeated T1 and ASL acquisitions.

FLAIR scans were assessed for white matter lesions by a consultant neuroradiologist, who gave their opinion on if they appeared clinically suspicious. White matter lesions are common imaging findings which can occur due to a variety of conditions and in normal ageing. Assessment of their clinical relevance is therefore best performed by an expert who is able to take into account their number and size with respect to subject age.

The MRS sequence is the same as one which is used in routine monitoring of all patients who attend the Sheffield Ataxia Centre, including patients with gluten ataxia in whom these readings are sensitive to dietary success[4]. In this clinical context there are local cut-off points for the N-Acetylaspartate/Creatine (NAA/Cr) ratio to indicate an abnormal scan (≤0.95). Replicating clinical procedure, NAA/Cr vermis values were ascertained from the scanner and compared to these limits to determine abnormal results.

To investigate for CBF change due to the gluten intervention, the average CBF for the brain’s grey matter (GM) was quantified from the pre- and post-gluten scans. ASL and M0 images were converted from DICOM to Nifti format using “dcm2niix” in a pipeline which ignored Philip’s pixel scaling and rescaling factors[5]. These were then processed using the FSL tool BASIL[6]. In addition to those factors already detailed for the core sequence, this processing included the following pertinent variables: independent structural data (including a skull-stripped image) was given using the same-acquisition T1 scan after bias-field correction using “N4”[7], the M0 image underwent “voxelwise” calibration, arterial transit time=1.3s, T1=1.3s, T1b=1.65s, inversion efficiency=0.85, and the final model implemented adaptive spatial regularization, a fixed label duration, partial volume correction and motion correction. Processed files were visually inspected for data quality and image registration success. CBF values used were those automatically calculated for GM given in the output file “perfusion_calib_gm_mean.txt”.

References

1. Currie S, Hadjivassiliou M, Clark MJ, et al. Should we be “nervous” about coeliac disease? Brain abnormalities in patients with coeliac disease referred for neurological opinion. J Neurol Neurosurg Psychiatry 2012;83(12):1216–21.

2. Hadjivassiliou M, Croall ID, Zis P, et al. Neurologic Deficits in Patients With Newly Diagnosed Celiac Disease Are Frequent and Linked With Autoimmunity to Transglutaminase 6. Clin Gastroenterol Hepatol 2019;17(13):2678–86.

3. Addolorato G, Di Giuda D, De Rossi G, et al. Regional cerebral hypoperfusion in patients with celiac disease. Am J Med 2004;116(5):312–7.

4. Hadjivassiliou M, Grunewald RA, Sanders DS, Shanmugarajah P, Hoggard N. Effect of gluten-free diet on cerebellar MR spectroscopy in gluten ataxia. Neurology 2017;

5. Chenevert TL, Malyarenko DI, Newitt D, et al. Errors in quantitative image analysis due to platform-dependent image scaling. Transl Oncol 2014;7(1):65–71.

6. Smith SM, Jenkinson M, Woolrich MW, et al. Advances in functional and structural MR image analysis and implementation as FSL. Neuroimage 2004;23(Suppl 1):S208-19.

7. Tustison NJ, Avants BB, Cook PA, et al. N4ITK: improved N3 bias correction. IEEE Trans Med Imaging 2010;29(6):1310–20.
